# *CYP3A4^∗^22* Genotyping in Clinical Practice: Ready for Implementation?

**DOI:** 10.3389/fgene.2021.711943

**Published:** 2021-07-08

**Authors:** Tessa A. M. Mulder, Ruben A. G. van Eerden, Mirjam de With, Laure Elens, Dennis A. Hesselink, Maja Matic, Sander Bins, Ron H. J. Mathijssen, Ron H. N. van Schaik

**Affiliations:** ^1^Department of Clinical Chemistry, Erasmus MC University Medical Center, Rotterdam, Netherlands; ^2^Department of Medical Oncology, Erasmus MC University Medical Center, Rotterdam, Netherlands; ^3^Integrated PharmacoMetrics, PharmacoGenomics and PharmacoKinetics, Louvain Drug Research Institute, Université Catholique de Louvain, Brussels, Belgium; ^4^Louvain Centre for Toxicology and Applied Pharmacology, Institut de Recherche Expérimentale et Clinique, Université Catholique de Louvain, Brussels, Belgium; ^5^Department of Internal Medicine, Erasmus MC University Medical Center, Rotterdam, Netherlands; ^6^Erasmus MC Transplant Institute, Rotterdam, Netherlands

**Keywords:** cytochrome P450, CYP3A4, *CYP3A4^∗^22*, genotyping, genotype-guided dosing, rs35599367, pharmacogenetics, personalized medicine

## Abstract

Cytochrome P450 3A4 (CYP3A4) is the most important drug metabolizing enzyme in the liver, responsible for the oxidative metabolism of ∼50% of clinically prescribed drugs. Therefore, genetic variation in *CYP3A4* could potentially affect the pharmacokinetics, toxicity and clinical outcome of drug treatment. Thus far, pharmacogenetics for CYP3A4 has not received much attention. However, the recent discovery of the intron 6 single-nucleotide polymorphism (SNP) rs35599367C > T, encoding the *CYP3A4^∗^22* allele, led to several studies into the pharmacogenetic effect of *CYP3A4^∗^22* on different drugs. This allele has a relatively minor allele frequency of 3-5% and an effect on CYP3A4 enzymatic activity. Thus far, no review summarizing the data published on several drugs is available yet. This article therefore addresses the current knowledge on *CYP3A4^∗^22*. This information may help in deciding if, and for which drugs, *CYP3A4^∗^22* genotype-based dosing could be helpful in improving drug therapy. *CYP3A4^∗^22* was shown to significantly influence the pharmacokinetics of several drugs, with currently being most thoroughly investigated tacrolimus, cyclosporine, and statins. Additional studies, focusing on toxicity and clinical outcome, are warranted to demonstrate clinical utility of *CYP3A4^∗^22* genotype-based dosing.

## Introduction

Cytochrome P450 enzymes (CYP450s) are responsible for the oxidative metabolism of many drugs, the most abundant enzyme being CYP3A4, and are involved in the metabolism of 50% of prescribed drugs (Wrighton et al., 2000; Danielson, 2002). Although analyzing genetic variation in *CYP*-genes is well known and nowadays used in clinical practice (Lauschke et al., 2017; Roden et al., 2019; van Schaik et al., 2020), single-nucleotide polymorphisms (SNPs) were thought to have a limited contribution to the observed variability in CYP3A4 activity, because of the unimodal distribution of enzyme activity (Lin et al., 2002) and the wide range of hepatic protein expression (Lamba et al., 2002a). Genetic variants changing amino acids are rare for *CYP3A4* (Lamba et al., 2002b). The relatively abundant *CYP3A4^∗^1B* variant (3–5%) (van Schaik et al., 2000) has been associated with altered drug metabolism (Rebbeck et al., 1998; Westlind et al., 1999), but results are inconsistent and its function remains controversial (Rebbeck et al., 1998; Amirimani et al., 1999; Ball et al., 1999; García-Martín et al., 2002; Lamba et al., 2002b; Spurdle et al., 2002; Wojnowski and Kamdem, 2006). This could be due to linkage disequilibrium with *CYP3A5^∗^1* (Zeigler-Johnson et al., 2004; Miao et al., 2009), suggesting that expression of CYP3A5 due to presence of *CYP3A5^∗^1* accounts for the association between CYP3A metabolic activity and *CYP3A4^∗^1B* (Kuehl et al., 2001). However, in 2011 the *CYP3A4* intron 6 SNP (rs35599367C > T, *CYP3A4^∗^22*) was described by Wang et al. (2011), using an allelic expression imbalance approach, explaining 12% of CYP3A4 enzyme activity variability. *CYP3A4^∗^22* predominantly occurs in Europeans and admixed Americans, compared to Africans and Asians (MAF: 5%, 2.6%, < 0.1%, < 0.6%, respectively) (Elens et al., 2011b; Zhou et al., 2017), and proved to encode decreased mRNA, protein and enzymatic activity *in vivo* (Wang et al., 2011; Klein et al., 2012; Elens et al., 2013b; Okubo et al., 2013). In *CYP3A4^∗^22*, formation of a non-functional alternative splice variant (aSV) was >100% increased, with partial intron 6 retention (Wang and Sadee, 2016). Interestingly, CYP3A4 aSV was found in liver, but not in small intestine (Wang and Sadee, 2016). Since its discovery, *CYP3A4^∗^22* effects have been described in several studies, but this information has not yet been summarized. This review addresses current knowledge on *CYP3A4^∗^22*, focusing on clinical studies. Unless otherwise indicated, statements on *CYP3A4^∗^22* reflect comparisons to *CYP3A4^∗^1/^∗^1* wild-type patients.

## Gold Standard CYP3A4 Phenotyping Probes: Midazolam and Erythromycin

Elens et al. (2013b) used the CYP3A golden standard phenotyping probes midazolam and erythromycin as indicators for the *in vivo* effect of *CYP3A4^∗^22* on drug metabolism, showing that cancer patients carrying *CYP3A4^∗^22* had a 40% reduction in erythromycin clearance and a 21% lower midazolam metabolic ratio (MR) (Elens et al., 2013b) (Supplementary Table 1). This indicates that *CYP3A4^∗^22* has potential clinically relevant effects.

## Immunosuppressive Agents

Cyclosporine and tacrolimus are immunosuppressants used in solid organ transplantation, metabolized by CYP3A4 into less active compounds, and are characterized by highly variable pharmacokinetics and a narrow therapeutic index (Moes et al., 2014). To prevent overexposure (risking drug-related toxicity) or underexposure (risking transplant rejection), therapeutic drug monitoring is applied (Kahan et al., 2002; Lloberas et al., 2017). Pharmacogenetic testing may optimize the starting dose when pharmacokinetic steady states are not yet achieved.

### Tacrolimus

Supra-therapeutic tacrolimus exposure is associated with toxicity (Miano et al., 2020), the main pharmacogenetic contributor being *CYP3A5*, first described by Hesselink et al. (2003). In 2011, Elens et al. (2011b) reported the influence of *CYP3A4^∗^22*, showing that *CYP3A4^∗^22* carriers have 16–76% increased tacrolimus pre-dose concentrations (C_0_) (Elens et al., 2011b; Elens et al., 2013a; Guy-Viterbo et al., 2014; Pallet et al., 2015) and 29–100% increased dose-adjusted tacrolimus C_0_ (C_0_/D) (Elens et al., 2011b,c, 2013a; Kurzawski et al., 2014; Pallet et al., 2015; Lloberas et al., 2017; Gómez-Bravo et al., 2018) (Supplementary Table 2). Combining *CYP3A4* and *CYP3A5* genotype into poor, intermediate and extensive CYP3A metabolizers explains >60% of tacrolimus C_0_/D variability (Elens et al., 2011c). Four kidney transplant recipients with [*CYP3A4^∗^22/^∗^22* + *CYP3A5^∗^3/^∗^3*] genotype showed a 91% increase in median tacrolimus C_0_/D compared to [*CYP3A4^∗^1/^∗^1* + *CYP3A5^∗^3/^∗^3*] patients (3.05 vs. 1.60 ng/ml/mg, respectively) and a 342% increase compared to [*CYP3A4^∗^1/^∗^1* + *CYP3A5^∗^1/^∗^1*] individuals (3.05 vs. 0.69 ng/ml/mg, respectively) (Scheibner et al., 2018). After 12 months, *CYP3A4^∗^22* carriers had 36.8% lower mean steady-state clearance and 50% lower dose requirements (de Jonge et al., 2015), comparable to other studies with 30–33% lower mean tacrolimus dose requirement for *CYP3A4^∗^22* carriers (Elens et al., 2011b; Gijsen et al., 2013; Pallet et al., 2015). Using a dosing algorithm, including *CYP3A4^∗^22* and *CYP3A5^∗^3* amongst other covariables, 58% of patients were on tacrolimus target at day 3 after transplantation (Francke et al., 2021) compared to 18.5–37.4% after initial bodyweight-based dosing (Thervet et al., 2010; Budde et al., 2014; Shuker et al., 2016). However, not all studies found a significant effect, but trends were observed (Santoro et al., 2013; Tavira et al., 2013; Lunde et al., 2014; Moes et al., 2014; Pulk et al., 2015; Debette-Gratien et al., 2016; Moes et al., 2016; Calvo et al., 2017; Madsen et al., 2017), which is possibly explained by low numbers of *CYP3A4^∗^22* carriers or population stratification differences.

### Cyclosporine

Cyclosporine treatment is mainly hampered by nephrotoxicity, limiting its clinical use (Kuroyanagi et al., 2018; Wu et al., 2018). In *CYP3A4^∗^22* carriers, cyclosporine C_0_/D were 60% higher (Elens et al., 2011c) (Supplementary Table 2). Combining *CYP3A4* and *CYP3A5* genotypes, poor CYP3A-metabolizers presented cyclosporine C_0_/D 54% and 114% higher compared to intermediate and extensive CYP3A-metabolizers, respectively (Elens et al., 2011c). Two other studies could not confirm this correlation, possibly due to a smaller patient population [*n* = 47, (Cvetković et al., 2017)], whereas Debette-Gratien et al. studied 170 liver transplant recipients (Debette-Gratien et al., 2016). For *CYP3A4^∗^22* carriers, a 53% higher dose-adjusted cyclosporine peak concentration (C_2_/D) (Lunde et al., 2014) and 15% lower cyclosporine clearance (Moes et al., 2014) were found. In total, 12% of interindividual variability in C_2_/D was explained by *CYP3A4^∗^22* carriership and Lunde et al. (2014) estimated that recipients carrying one *CYP3A4^∗^22* allele would need 50% less cyclosporine to reach therapeutic targets. Additionally, a significant association between *CYP3A4^∗^22* and mean difference in second and first dose was found (El-Shair et al., 2019). Mixed-model analysis showed a 20% lower overall creatinine clearance, indicating cyclosporine-induced nephrotoxicity in *CYP3A4^∗^22* patients receiving cyclosporine/mycophenolate mofetil (Elens et al., 2012). Clinically, *CYP3A4^∗^22* carriers showed a significantly higher risk of delayed graft function, with an odds ratio (OR) of 6.34 (Elens et al., 2012).

### Everolimus and Sirolimus

Everolimus and sirolimus, immunosuppressive drugs inhibiting activation and proliferation of T-lymphocytes through mammalian Target Of Rapamycin (mTOR), are used to prevent transplant rejection as tacrolimus and cyclosporine-sparing agents (Cravedi et al., 2010; Moes et al., 2012; Woillard et al., 2013). Both drugs are metabolized by CYP3A4 and CYP3A5 into less active compounds (Woillard et al., 2013; Pascual et al., 2017). A 20% lower sirolimus metabolism was found in human liver microsomes of *CYP3A4^∗^22* carriers (Woillard et al., 2013), whereas for everolimus, a trend of 7% lower clearance in *CYP3A4^∗^22* carriers was observed (Moes et al., 2014) (Supplementary Table 2). Metastatic breast cancer patients carrying *CYP3A4^∗^22* had 170% higher everolimus plasma concentrations (Pascual et al., 2017). It is important to realize that patients with metastatic breast cancer receive considerably higher doses than solid organ transplant recipients (Shipkova et al., 2016).

## Cardiology

Ticagrelor, clopidogrel, and prasugrel are active platelet aggregation inhibitors metabolized by CYP3A4, used in cardiology and neurology. For ticagrelor, *CYP3A4^∗^22* carriers showed an 89% higher area-under-the-plasma-concentration-time curve (AUC) and more pronounced platelet inhibition (43% vs. 21%) (Holmberg et al., 2019) (Supplementary Table 3). This increases risk of bleedings (Becker et al., 2011). No significant correlation of *CYP3A4^∗^22* with active metabolites of clopidogrel or prasugrel was found, although the authors stated that differences in clopidogrel and prasugrel pharmacokinetics <50% cannot be ruled out (Holmberg et al., 2019). For sildenafil, metabolized by CYP3A4 in less active compounds, is used in patients with heart failure, *CYP3A4^∗^22* carriers showed increased dose-adjusted concentrations (de Denus et al., 2018). For these drugs, only one study is currently available. Further studies are needed to confirm these findings.

For statins, *CYP3A4^∗^22* carriers required significantly lower doses for optimal lipid control (Wang et al., 2011). Simvastatin and atorvastatin are primarily metabolized by CYP3A4 into less active metabolites (Vickers et al., 1990; Prueksaritanont et al., 2003). *CYP3A4^∗^22* carriers demonstrated 49% higher simvastatin bioavailability (Tsamandouras et al., 2014), resulting in 20% higher simvastatin plasma concentrations (Kitzmiller et al., 2014) and 58% increased plasma 12-h concentrations (Luzum et al., 2015) (Supplementary Table 3). For atorvastatin, healthy *CYP3A4^∗^22* carriers showed 35% decreased MR, confirming lower CYP3A4 activity (Klein et al., 2012). The 2-hydroxyatorvastatin/atorvastatin AUC_*in*__*f*_ ratio was 35% lower per copy of *CYP3A4^∗^22* (Klein et al., 2012). *CYP3A4^∗^22* carriers showed a greater reduction in total- and LDL-cholesterol levels (-0.31 and -0.34 mmol/l), consistent with expected higher simvastatin plasma concentrations, in Dutch Caucasians (Elens et al., 2011a). This finding could not be confirmed by Ragia et al. (2015) in Greek patients with hypercholesterolemia (*n* = 416), although the authors stated that potential confounders required further studies.

## Psychiatry

### Antidepressants

*CYP2D6* pharmacogenetic based guiding of antidepressant drug therapy is nowadays quite accepted. Dosing advices, based on *CYP2D6* and *CYP2C19* genotype, are available at PharmGKB, CPIC and the Dutch Pharmacogenetic Working Group (DPWG) (Hicks et al., 2015). Thus far, *CYP3A4* genotype-based dosing for psychotropic drugs has not yet been described. However, CYP3A4 does have a contribution in citalopram [CYP2C19, CYP2D6, CYP3A4 (Rochat et al., 1997)], escitalopram [CYP2C19, CYP3A4, CYP2D6 (von Moltke et al., 2001)] and mirtazapine [CYP2D6, CYP3A4, CYP1A2 (Störmer et al., 2000)] metabolism. The prominent role of CYP2C19 and CYP2D6 in the metabolism may complicate CYP3A4 studies in these drugs. Yet, compound heterozygotes for *CYP2D6*, *CYP2C19*, and *CYP3A4* were found by us in patients with therapy related side effects, insufficiently explained by *CYP2D6* and/or *CYP2C19* genotype alone. Recently, combining CYP2C19, CYP2D6, and CYP3A4 genotypes proved to be a better predictor of citalopram/escitalopram blood levels as compared to individual genes (Shelton et al., 2020). We feel it would be beneficial to study the effect of *CYP3A4^∗^22*, in addition to *CYP2D6* and *CYP2C19*, in antidepressants, but thus far, no clear indications for a prominent role for *CYP3A4^∗^22* are available.

### Anti-anxiolytics

Alprazolam is one of the most commonly prescribed psychoactive agent for mood and anxiety disorders (Stahl, 2002). In patients with alcoholism and anxiety disorders, *CYP3A4^∗^22* carriers had significantly increased active alprazolam concentration/dose ratios and a decreased treatment response, as reflected in HAMA scale scores (4.0 vs. 3.0) (Zastrozhin et al., 2020) (Supplementary Table 4).

### Anti-psychotics

Risperidone, a drug with *CYP2D6* genotype-based dosing recommendations, showed *CYP3A4^∗^22* carriers having 30% lower 9-hydroxyrisperidone (active metabolite, generated from risperidone by CYP2D6) clearance (Vandenberghe et al., 2015). Two other studies could not confirm this: the reason for this discrepancy could be sample size [*n* = 26, (Rafaniello et al., 2018)], although van der Weide et al. included 130 patients (van der Weide and van der Weide, 2015) (Supplementary Table 5). *CYP3A4^∗^22* genotype was associated with serum levels active pimozide and C/D ratio, however, this association only explained 5% of total variation in a multiple regression analysis (van der Weide and van der Weide, 2015). For aripiprazole and haloperidol, no significant effect of *CYP3A4^∗^22* was observed (van der Weide and van der Weide, 2015; Rafaniello et al., 2018), possibly because CYP2D6 plays a more prominent role (Fang et al., 1997; Fang et al., 1999; van der Weide and van der Weide, 2015). Multiple regression analysis demonstrated that 4–17% of the variation in concentration of these anti-psychotics was explained by *CYP2D6* genotype (van der Weide and van der Weide, 2015). However, quetiapine, a drug metabolized by CYP3A4 (DeVane and Nemeroff, 2001), showed 150% higher serum concentrations and 67% higher dose-corrected quetiapine serum concentrations in *CYP3A4^∗^22* carriers (van der Weide and van der Weide, 2014). Significantly more *CYP3A4^∗^22* patients achieved serum levels above the therapeutic range of 500 μg/L (van der Weide and van der Weide, 2014).

## Anticancer Agents

For anticancer drugs, pharmacogenetic testing for CYP450 enzymes is currently limited to *CYP2D6* testing for tamoxifen, which needs activation by CYP2D6 [for review, see Mulder et al. (2021)]. A good example of implementation of pharmacogenetic testing in oncology outside the CYP450 field, is *DPYD* analysis prior to capecitabine treatment, as well as *TPMT* testing for 6-mercaptopurine for treatment of Acute Lymphatic Leukemia (Henricks et al., 2018; Roden et al., 2019). Yet, CYP3A4 is involved in the metabolism of many anticancer drugs, and since efficacy and toxicity are important aspects in oncology, factors that may contribute to predicting toxicity should be examined for their potential clinical value. As indicated by Elens et al., decreased CYP3A4 metabolism due to *CYP3A4^∗^22* could be demonstrated in cancer patients, as determined by midazolam and erythromycin metabolism (Elens et al., 2013b).

### Hormonal Treatment: Tamoxifen and Exemestane

Tamoxifen, standard-of-care in (adjuvant) treatment of ER-positive breast cancer patients (FDA, 1977; Jordan, 2014), is metabolized by CYP3A4 into inactive N-desmethyltamoxifen. This is converted by *CYP2D6* to the active endoxifen. While *CYP2D6* contributes most to variation in systemic exposure to endoxifen (Goetz et al., 2018; Puszkiel et al., 2020), *CYP3A4^∗^22* also affects this variation: *CYP3A4^∗^22* carriers showed higher tamoxifen and 4-hydroxytamoxifen (Teft et al., 2013; Antunes et al., 2015), but also higher N-desmethyltamoxifen and endoxifen concentrations (Teft et al., 2013; Baxter et al., 2014) (Supplementary Table 6). Despite increased tamoxifen and endoxifen exposure, *CYP3A4^∗^22* carriers were significantly less likely to experience hot flashes (OR: 8.87) (Baxter et al., 2014). Since these studies show conflicting results, no clear conclusions can be drawn and further research is needed. Exemestane, another drug used in breast cancer treatment, is also metabolized by CYP3A4. For this drug, 54% higher steady-state active exemestane concentrations were found in *CYP3A4^∗^22* carriers (Hertz et al., 2017).

### Microtubule-Stabilizing Agents: Paclitaxel and Docetaxel

Paclitaxel and docetaxel are used for solid tumors. For paclitaxel, CYP2C8 has been the focus for CYP450 studies, but also CYP3A4 is involved in its metabolism (Harris et al., 1994). Neurotoxicity is often observed as side effect (Lee and Swain, 2006). In 261 cancer patients, *CYP3A4^∗^22* carrier status was an independent predictive factor for development of paclitaxel-induced neurotoxicity, although no significant association with paclitaxel pharmacokinetics was found for reasons unknown to the authors (Supplementary Table 6) (de Graan et al., 2013). Interestingly, another *CYP3A4* variant allele, *CYP3A4^∗^20*, was also associated with paclitaxel neuropathy (Apellániz-Ruiz et al., 2015). This supports the potential predictive value of *CYP3A4* genotyping for paclitaxel neurotoxicity. For docetaxel, a study in 150 breast cancer patients showed that *CYP3A4^∗^22* carriers were at increased risk of grade 3-4 toxicity (Sim et al., 2018). Docetaxel is mainly metabolized by CYP3A4 (Shou et al., 1998; Hirth et al., 2000), and large interindividual variability in docetaxel pharmacokinetics has been reported (Goh et al., 2002; Michael et al., 2012). Since the pharmacokinetic association of *CYP3A4^∗^22* is lacking, more research on *CYP3A4^∗^22* and docetaxel treatment is warranted.

### Tyrosine Kinase Inhibitors: Sunitinib and Pazopanib

Sunitinib and pazopanib are frequently prescribed tyrosine kinase inhibitors (TKIs) with established exposure-response relationships for renal cell carcinoma (Houk et al., 2010; Suttle et al., 2014). Both drugs are predominantly metabolized by CYP3A4 into less active metabolites (Sugiyama et al., 2011; Thorn et al., 2017). *CYP3A4^∗^22* status was associated with a significantly decreased clearance of pazopanib (35%) (Bins et al., 2019) and a decreased clearance of sunitinib (22.5%) (Diekstra et al., 2014) (Supplementary Table 6). Bins et al. (2019) proposed that pazopanib dose adjustments based on *CYP3A4^∗^22* status should be considered since their pharmacokinetic-model showed that 600 mg pazopanib in *CYP3A4^∗^22* carriers would lead to similar pazopanib exposure as wild-type patients using 800 mg. Feasibility of *CYP3A4^∗^22* genotype-guided dosing of TKIs in cancer patients is currently under investigation in a large prospective clinical trial; results are expected in 2022 (Dutch Trial Registry^1^).

## Pain Medication: Fentanyl

Synthetic opioids, like alfentanil, sufentanil, remifentanil, and fentanyl, are all metabolized by CYP3A4 into inactive metabolites (Yun et al., 1992; Tateishi et al., 1996). Unfortunately, no studies regarding the association between *CYP3A4^∗^22* and pharmacokinetics of alfentanil, sufentanil, and remifentanil are published. Regarding fentanyl, a positive association between *CYP3A4^∗^22* carriers and increased exposure to fentanyl (higher AUC, lower clearance) was found in two studies with healthy individuals (Saiz-Rodríguez et al., 2019; Saiz-Rodríguez et al., 2020) (Supplementary Table 7). An association with fentanyl MR confirmed this influence, although the authors did not find a significant association with serum fentanyl concentration (Barratt et al., 2014).

## Anti-Viral Agents

Lopinavir and tenofovir alafenamide are metabolized by CYP3A4 into less active metabolites. Lopinavir is co-administered with ritonavir as a booster drug to enhance its oral bioavailability and plasma half-life by, in fact, inhibiting CYP3A4 activity (Ernest et al., 2005; Olagunju et al., 2014). In the presence of ritonavir-induced CYP3A4 inhibition, in 375 HIV-positive patients using 400/100 mg lopinavir/ritonavir, *CYP3A4^∗^22/^∗^22* patients had lower lopinavir clearances compared to *CYP3A4^∗^1/^∗^1* patient and compared to *CYP3A4^∗^22* carriers (Olagunju et al., 2014). This resulted in 130% higher lopinavir C_0_ (Olagunju et al., 2014) (Supplementary Table 8). A trend of lower lopinavir clearance in *CYP3A4^∗^22* carriers was also observed (Olagunju et al., 2014). *CYP3A4^∗^22* carriers also had a 39% higher plasma tenofovir alafenamide AUC_0__–__24_ at day 56 (Cerrone et al., 2019) in patients receiving tenofovir alafenamide/emtricitabine in co-administration of rifampicin, a known CYP3A4-inducer (Niemi et al., 2003).

## Conclusion

Although high interindividual variability is observed in CYP3A4 expression and activity, this could not be accounted for by genetic variability in *CYP3A4*, as polymorphisms that result in a significant altered CYP3A4 activity are rare. Therefore, the discovery that the more common observed *CYP3A4^∗^22* has significant effects on the pharmacokinetics of several drugs was surprising. This argues in our opinion for further investigation of the potential clinical use of *CYP3A4^∗^22* genotyping (Table 1), especially for tacrolimus, cyclosporine, midazolam and erythromycin for which the effects are strong. Ticagrelor, alprazolam, quetiapine, fentanyl, lopinavir, and various anticancer drugs should be considered, since several publications demonstrate effects of *CYP3A4^∗^22* carrier status. For several drugs, like tacrolimus, tamoxifen, antidepressants, and antipsychotics, *CYP3A4^∗^22* should be considered only in combination with other *CYP* genotyping analyses to generate a complete picture on CYP450 status. Since CYP3A4 is highly subject to food and drug induction/inhibition, this should also be taken into account. Effects of *CYP3A4^∗^22* on simvastatin treatment were confirmed by several studies, yet the clinical sue seems limited due to easy effect measurement (cholesterol levels). For the purpose of this review, we have focused on clinical studies related to pharmacokinetics, and the discussion of *in vitro* investigation is not exhaustive. Correlations with clinical effects of *CYP3A4^∗^22* regarding treatment toxicity or efficacy would need further investigation. Seeing the relations for *CYP3A4^∗^22* on pharmacokinetics of several drugs, this seems a logical next step.

**TABLE 1:** Summary of effect of CYP3A4*22 on pharmacokinetics (PK), dose requirement (DR), toxicity (Tox) or effect (Eff) of the described drugs.

Drug class	Drug	CYP3A4-guided dosing?	Effect of CYP3A4*22				Studies that show		
			PK	DR	Tox	Eff	Sign. effect	Trends	No effect or opposite effect
*CYP3A4 phenotyping probes*	Erythromycin	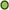	N-des-methylation activity ↓				PK: 1	PK: 0	PK: 0
	Midazolam (MDZ)	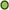	1’-OH-MDZ:MDZ ↓				PK: 1	PK: 0	PK: 0
*Immunosuppressants*	Tacrolimus	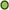	C0/D ↑	D↓			PK: 9 D: 5	PK: 7 D: 0	PK: 3 D: 0
	Cyclosporine	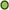	C0/D ↑		OR delayed graft function ↑		PK: 3 D: 1 Tox: 1	PK: 0 D: 0 Tox: 1	PK: 3 D: 0 Tox: 0
	Sirolimus	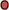	*In vitro* metabolic rate ↓				PK: 1	PK: 0	PK: 0
	Everolimus	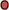	C ↑*				PK: 1	PK: 1	PK: 0
*Cardiology*	Ticagrelor	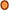	AUC ↑		Platelet inhibition ↑	PK: 1 Eff: 1	PK: 0 Eff: 0	PK: 0 Eff: 0	
	Sildenafil	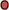	Cmax/D ↑				PK: 1	PK: 0	PK: 0
	Simvastatin	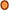	C ↑	D↑		Total and LDL cholesterol ↑	PK: 3 D: 1 Eff: 1	PK: 1 D: 0 Eff: 0	PK: 0 D: 0 Eff: 0
	Atorvastatin	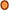	2-OH- atorvastatin/atorvastatin AUCinf ↓				PK: 1 D: 1	PK: 1 D: 0	PK: 0 D: 0
*Anxiolytics*	Alprazolam	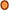	C:D ↑			HAMA scale score ↑	PK: 1 Eff: 1	PK: 0 Eff: 0	PK: 0 Eff: 0
*Anti-psychotics*	Risperidone	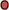	Cl ↓				PK: 1	PK: 2	PK: 0
	Pimozide	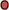	Cl ↓				PK: 1	PK: 0	PK: 0
	Quetiapine	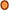	C/D ↑				PK: 1	PK: 0	PK: 0
*Anticancer drugs*	Tamoxifen	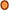	C 4-OH-tam↑ C endoxifen ↑ C tam ↑		OR hot flash ↓		PK: 1 Tox: 1	PK: 0 Tox: 0	PK: 0 Tox: 0
	Exemestane	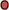	Css ↑				PK: 1	PK: 0	PK: 0
	Paclitaxel	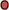			OR neuro- toxicity↑		Tox: 1	Tox: 0	Tox: 0
	Docetaxel	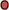			OR grade 3-4 toxicity↑		Tox: 1	Tox: 0	Tox: 0
	Pazopanib	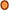	Cl ↓	D↓			PK: 1 D: 1	PK: 0 D: 0	PK: 0 D: 0
	Sunitinib	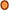	Cl ↓ *				PK: 0	PK: 1	PK: 0
*Analgesia*	Fentanyl	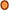	Norfentanyl: fentanyl ratio ↓				PK: 2	PK: 1	PK: 0
*Anti-viral drugs*	Lopinavir	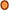	C0↑	D↓			PK: 1 D: 1	PK: 0 D: 0	PK: 0 D: 0
	Tenofovir alafenamide	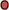	AUC ↑				PK: 1	PK: 0	PK: 0

Third column shows our opinion on the level of evidence considering CYP3A4*22 based dosing in corresponding drug treatment [green: sufficient evidence, orange: more evidence is needed to fully establish clinical value, red: too limited (non-significant) evidence]. Trends are marked with an asterisk (*). AUC, area under the plasma concentration-time curve; C, plasma concentration (not specified); C0, trough concentration; C0/D, dose-adjusted trough concentration; Cl, Clearance; Css, steady-state concentration; D, required dose; OR: odds ratio.

## Author Contributions

TM wrote the first draft of the manuscript and made all Supplementary Tables. TM and RE made Table 1. Supervision by RS. RE, MW, LE, DH, MM, SB, RM, and RS wrote sections of the manuscript and collected relevant references. All authors contributed to manuscript revision, read, and approved the submitted version.

## Conflict of Interest

The authors declare that the research was conducted in the absence of any commercial or financial relationships that could be construed as a potential conflict of interest.

## Footnotes

1 https://www.trialregister.nl/trial/7514
