# Supplementary material for: CYP3A4∗22 Genotyping in Clinical Practice: Ready for Implementation?
Source: Front Genet. 2021 Jul 8;12:711943. doi: 10.3389/fgene.2021.711943 (PMC8296839; doi:10.3389/fgene.2021.711943)
Supplement: Supplementary file 1 [file Table_1.docx]

Supplementary Table 1

*CYP3A4*22* Genotyping in Clinical Practice: Ready for Implementation?

*Tessa A.M. Mulder, Ruben A. G. van Eerden, Mirjam de With, Laure Elens, Dennis A. Hesselink, Maja Matic, Sander Bins, Ron H. J. Mathijssen and Ron H. N. van Schaik*

| **Supplementary Table 1: Summary of CYP3A4*22 influence on pharmacokinetics of CYP3A4 phenotyping probes erythromycin and midazolam.** | | | | |
| --- | --- | --- | --- | --- |
| **Drug** | **n=** | **Study population** | **Estimated change** | **Reference** |
| Erythromycin | 45 | Predominantly Caucasian patients with advanced solid tumors treated with chemotherapy in previous trials | Erythromycin N-demethylation activity, measured by erythromycin breath test, was 40% lower in *CYP3A4*22* carriers compared to wild type patients (p=0.032). | (Elens, 2013b) |
| Midazolam (MDZ) | 108 | Predominantly Caucasian patients with advanced solid tumors treated with chemotherapy in previous trials | 20.7% lower 1’-OH-MDZ:MDZ in *CYP3A4*22* carriers compared to *CYP3A4*1/*1* patients (p=0.01). 38.7% decrease in 1’-OH-MDZ:MDZ in poor (*CYP3A4*22-CYP3A5*3/*3*) compared with extensive (*CYP3A4*1/*1-CYP3A5*1*) CYP3A metabolizers (p<0.001). | (Elens, 2013b) |

**References**

Please see main article for references:
*Mulder TAM, van Eerden RAG, de With M, Elens L, Hesselink DA, Matic M, Bins S, Mathijssen RHJ and van Schaik RHN (2021) CYP3A4∗22 Genotyping in Clinical Practice: Ready for Implementation? Front. Genet. 12:711943. doi: 10.3389/fgene.2021.711943*
