# Supplementary material for: CYP3A4∗22 Genotyping in Clinical Practice: Ready for Implementation?
Source: Front Genet. 2021 Jul 8;12:711943. doi: 10.3389/fgene.2021.711943 (PMC8296839; doi:10.3389/fgene.2021.711943)
Supplement: Supplementary file 2 [file Table_2.docx]

Supplementary Table 2

*CYP3A4*22* Genotyping in Clinical Practice: Ready for Implementation?

*Tessa A.M. Mulder, Ruben A. G. van Eerden, Mirjam de With, Laure Elens, Dennis A. Hesselink, Maja Matic, Sander Bins, Ron H. J. Mathijssen and Ron H. N. van Schaik*

| **Supplementary Table 2: Summary of CYP3A4*22 influence on pharmacokinetics (PK), dose requirement (DR), or toxicity (Tox) of tacrolimus, cyclosporine, sirolimus, and everolimus. Abbreviations: AUC: area under the plasma concentration-time curve, C0: pre-dose concentration, C0/D: dose-adjusted trough concentration.** | | | | | | |
| --- | --- | --- | --- | --- | --- | --- |
| **Drug** | ***Effect*** | ***N=*** | ***Study***  ***population*** | ***Estimated change*** | ***Reference*** |  |
| Tacrolimus (Tac) | PK | 185 | Predominantly Caucasian kidney transplant recipients | 38% increased Tac C0 3 days after transplantation (p=0.05) in *CYP3A4*22* carriers compared to *CYP3A4*1/*1* patients (20.5 vs. 14.9 µg/l). 36% increased Tac C0/D 10 days after transplantation (p=0.006) and 56% increased Tac C0/D after one month (p=0.006) in *CYP3A4*22* carriers compared to *CYP3A4*1/*1* patients (101.3 vs. 74.3, and 136.6 vs 87.5 µg/l per mg/kg, respectively) | (Elens et al., 2011b) |  |
|  |  | 96 | Belgium kidney transplant recipients | 15.9% overall increase in Tac C0 (p<0.001), 27.7% increase in Tac C0 in the first 5 days after transplantation (p<0.001), 52% increase in Tac C0/D estimated mean (P<0.001) and 66,8% increase in dose-adjusted Tac whole blood concentrations (p<0.001) in *CYP3A4*22* carriers compared to *CYP3A4*1/*1* patients. | (Elens et al., 2013a) |  |
|  |  | 99 | Caucasian stable renal transplant patients | 2.0-fold higher Tac C0/D (0.017) in *CYP3A4*22* carriers compared to *CYP3A4*1/*1* patients. The CYP3A4/CYP3A5 poor metabolizer group had 1.6-fold higher Tac C0/D (p<0.001) compared to the intermediate metabolizer group, and 4.1-fold higher (p<0.001) than the extensive metabolizer group. Multiple linear regression analysis showed that both CYP3A4*22 and CYP3A5*3 SNPs explained more than 60% of the variability observed in dose adjusted tacrolimus C0. | (Elens et al., 2011c) |  |
|  |  | 89 | Spanish de novo liver graft recipients | 1.9-fold average increase in Tac C0/D at day 7 (p=0.047), day 14 (p=0.025) and day 30 (p=0.053) in *CYP3A4*22* carriers compared to *CYP3A4*1/*1* (all CYP3A5 non-expressers). | (Gómez-Bravo et al., 2018) |  |
|  |  | 241 | Polish Caucasian kidney transplantation patients | 28.8% increased Tac C0/D at 3 months (p=0.024) after transplantation in *CYP3A4*22* carriers compared to *CYP3A4*1/*1* (180.2 vs 139.9 ng/ml per mg/kg/day) | (Kurzawski et al., 2014) |  |
|  |  | 186 | Predominantly Caucasian kidney transplant recipients | 55.6% higher mean Tac C0 (p<0.001) at day 10 in *CYP3A4*22* carriers compared to *CYP3A4*1/*1* patients (23.5 vs. 15.1 ng/ml). Significantly higher Tac C0/D (p<0.001) in *CYP3A4*22* carriers than *CYP3A4*1/*1* (only figure available) | (Pallet et al., 2015) |  |
|  |  | 4 | Caucasian kidney transplant recipients | 90.6% increase in median Tac C0/D in *CYP3A4*22/*22-CYP3A5*3/*3* patients compared to *CYP3A4*1/*1-CYP3A5*3/*3* (3.05 vs 1.60 ng/ml/mg, respectively) and a 342% increase compared to *CYP3A4*1/*1-CYP3A5*1/*1* (3.05 vs. 0.69 ng/ml/mg, respectively). No p-values reported. | (Scheibner et al., 2018) |  |
|  |  | two cohorts: Tested at  3 months (n=59) and 1-5 years (n=80) | Caucasian renal allograft recipients | 36.8% decreased bodyweight-corrected tacrolimus mean steady-state oral Tac clearance (p=0.006) at >12 months after transplantation in *CYP3A4*22* carriers compared to *CYP3A4*1/*1* patients. | (de Jonge et al., 2015) |  |
|  |  | 114 | Predominantly Caucasian pediatric liver transplant patients | Donor CYP3A4*22 was significantly associated with higher Tac C0 at day 3 and 10, compared to donor CYP3A4*1/*1 (22.4 ± 16.9 μg/l vs 12.7 ± 6.6; p = 0.035 at day 3, and 14.9 ± 7.1 vs 8.9 ± 3.4 μg/l; p = 0.015 at day 10). There was no significant effect of recipient CYP3A4 genotype on Tac PK. | (Guy-Viterbo et al., 2014) |  |
|  |  | 272 | Kidney transplant recipients | Poor CYP3A metabolizers [CYP3A4*22 carrier+CYP3A5*3/*3] had 26% higher Tac C0/D compared to intermediate metabolizers [CYP3A4*1/*1+CYP3A5*3/*3] or [CYP3A4*22 carrier+CYP3A5*1 carrier] (p<0.001). Extensive metabolizers had 88% lower Tac C0/D compared with intermediate metabolizers (p<0.001). | (Lloberas et al., 2017) |  |
|  |  | 140 | Brazilian renal transplant Patients | Non-significant increase in Tac C0/D in *CYP3A4*22* carriers compared to *CYP3A4*1/*1* patients (1.48±0.69 vs. 1.19±0.83, p=0.34) | (Santoro et al., 2013) |  |
|  |  | 206 | White (from Asturias, northern Spain) | Non-significant difference in total Tac normalized (ng/ml per mg/day) after 1 week (1.27 (95% CI 0.40-3.68) vs. 1.16 (95% CI 0.27-7.07), p=0.57) and 6 months (1.85 (95% CI 0.71-13) vs. 1.90 (95% CI 0.50-12.33, p=0.98) in *CYP3A4*22* carriers compared to *CYP3A4*1/*1* patients. | (Tavira et al., 2013) |  |
|  |  | 117 | Norwegian renal transplant recipients | Non-significant small increase in Tac C0/D and less variance in patients carrying *CYP3A4*22* compared to *CYP3A4*1/*1* patients (see Ref., Figure 1). | (Lunde et al., 2014) |  |
|  |  | 1407 | Predominantly Caucasian and African-American kidney transplant recipients | In a multivariable model, the effect of *CYP3A4*22* on tacrolimus trough concentration is 0.044 (95% CI -0.038-0.125, p=0.29). | (Pulk et al., 2015) |  |
|  |  | 66 | Predominantly Caucasian stable liver transplant recipients | Not-significant 17% increase in mean tacrolimus clearance when the recipient is a CYP3A4*22 carrier (p=0.25) compared to the reference group. 0% change in mean tacrolimus clearance when the donor is a CYP3A4*22 carrier (p=0.53) | (Moes et al., 2016) |  |
|  |  | 67 | Predominantly Caucasian pediatric liver transplant recipients | Statistically different Tac C0 were detected between rapid, extensive and intermediate CYP3A4/5 metabolizers (4.1 ng/ml RM vs. 9.2 ng/ml EM, p<0.005, and 4.1 ng/ml RM vs. 8.5 ng/ml IM, p<0.02). No statistically significant difference in tacrolimus concentration/weight-adjusted dose ratio was found. | (Calvo et al., 2017) |  |
|  |  | 72 | Danish pediatric and adult kidney transplant recipients | At 6 weeks post-transplantation, CYP3A4*22 carriers had (non-significantly) 177% higher median tacrolimus C0/D compared with CYP3A4*1/*1 patients (p=0.10). No trend observed at 1 year post-transplantation. | (Madsen et al., 2017) |  |
|  |  | 101 | Predominantly Caucasian adult renal transplant recipients | A trend of 16% lower Tac clearance (p=0.22) in *CYP3A4*22* carriers compared to *CYP3A4*1/*1* patients. | (Moes et al., 2014) |  |
|  |  | 170 | French liver transplantation patients | Significant influence of recipient, and donor *CYP3A4*22* alleles on Tac log-transformed C0/D, however *CYP3A4*22* recipient and donor genotypes had opposite effects, leading to the absence of effect of the combined recipient and donor statuses on Tac C0/D (β=0.08±0.04, p=0.08). | (Debette-Gratien et al., 2016) |  |
|  | *In vivo* CYP3A4 enzyme activity | two cohorts: tested at 3 months (n=59) tested at 1-5 years (n=80) | Caucasian renal allograft recipients | 31.7-33.6% reduction in midazolam apparent oral clearance (reflecting reduced in vivo CYP3A4 activity) in CYP3A4*22 carriers compared to CYP3A4*1/*1 patients (all CYP3A5 non-expressers; p=0.04 and p-0.05, respectively). | (de Jonge et al., 2015) |  |
|  | DR | 185 | Predominantly Caucasian kidney transplant recipients | 33% lower overall mean daily-dose requirement (p=0.018) to reach the same predose Tac blood concentrations for *CYP3A4*22* carriers compared to *CYP3A4*1/*1* patients | (Elens et al., 2011b) |  |
|  |  | two cohorts: tested at 3 months (n=59) tested at 1-5 years (n=80) | Caucasian renal allograft recipients | 50% lower Tac mean dose requirements (2.7 vs 5.4 mg/day, p=0.0007) and 43% lower bodyweight-corrected dose requirement (0.04 mg/kg/day vs 0.07 mg/kg/day, p=0.0002) in *CYP3A4*22* carriers compared to *CYP3A4*1/*1.* | (de Jonge et al., 2015) |  |
|  |  | 89 | Spanish de novo liver graft recipients | 31.6% lower Tac dose requirements (p=0.026) at day 14 after transplantation (*CYP3A4*22* carriers 0.067 vs. *CYP3A4*1/*1* 0.098 mg/kg/day) | (Gómez-Bravo et al., 2018) |  |
|  |  | 186 | Predominantly Caucasian kidney transplant recipients | 33% lower Tac dose requirement for *CYP3A4*1/*22* patients compared to *CYP3A4*1/*1* patients (based on regression coefficient for the fixed effect *CYP3A4* (0.67), no p-value). | (Pallet et al., 2015) |  |
|  |  | 56 | Predominantly Caucasian pediatric heart transplant recipients | 30% lower dose requirement (p=0.016) for *CYP3A4*22* carriers compared to *CYP3A4*1/*1.* | (Gijsen et al., 2013) |  |
| Cyclosporine (CsA) | PK | 99 | Caucasian stable renal transplant patients | 1.6-fold higher CsA C0/D (p=0.019) in *CYP3A4*22* carriers compared to *CYP3A4*1/*1* patients. The CYP3A4/CYP3A5 poor metabolizer group had 1.5-fold higher CsA C0/D (p=0.012) compared to the intermediate metabolizer group, and 2.2-fold higher (p=0.006) than the extensive metabolizer group. Multiple linear regression analysis showed that both CYP3A4*22 and CYP3A5*3 SNPs explained more than 20% of the variability observed in dose adjusted tacrolimus C0. | (Elens et al., 2011c) |  |
|  |  | 35+19 | Norwegian renal transplant recipients | 53% higher dose adjusted concentration cyclosporine (C2/D)(p=0.03) in *CYP3A4*22* carriers compared to *CYP3A4*1/*1* patients. The CYP3A4*22 allele explained 12% of the interindividual variability in CsA C2/D ratio (r=0.35, p<0.01). | (Lunde et al., 2014) |  |
|  |  | 298 | Predominantly Caucasian adult renal transplant recipients | 15% lower CsA clearance (p<0.001) in *CYP3A4*22* carriers compared to *CYP3A4*1/*1* patients. | (Moes et al., 2014) |  |
|  |  | 170 | French liver transplantation patients | Non-significant influence of recipient (β=-0.06±0.07, p=0.40), donor (β=-0.07±0.09, p=0.40), and both donor and recipient (β=-0.08±0.06, p=0.18) *CYP3A4*22* alleles on CsA log-transformed C0/D. | (Debette-Gratien et al., 2016) |  |
|  |  | 47 | Serbian pediatric renal transplant recipients | Multivariate regression for the influence of CYP3A4*22 showed non-significant effect on overall CsA C0/D (β=0.10±0.14, p=0.69), and on overall C2/D (β=-0.09±0.11, p=0.87). | (Cvetković et al., 2017) |  |
|  |  | 109 | Jordanian adult kidney transplantation patients | No association between the mean of the first C2 blood levels in CYP3A4*22 carriers compared to CYP3A4*1/*1 patients (893.63 vs. 1287.10p=0.063) | (El-Shair et al., 2019) |  |
|  | DR | 109 | Jordanian adult kidney transplantation patients | Significant association between CYP3A4*22 and mean difference between the second and first given dose (p=0.034) in *CY3A4*22* carriers compared to *CYP3A4*1/*1* patients (mean difference 25.00 vs. -0.99, no units). | (El-Shair et al., 2019) |  |
|  | Tox | 172 | Predominantly White d*e-novo* kidney transplant recipients receiving CsA/mycophenolate mofetil | 20% lower overall creatinine clearance (p=0.002) in *CYP3A4*22* carriers compared to *CYP3A4*1/*1.* Higher risk of delayed graft function, odds ratio = 6.34 (p=0.015), for *CYP3A4*22* carriers compared to *CYP3A4*1/*1.* | (Elens et al., 2012) |  |
|  |  | 109 | Jordanian adult kidney transplantation patients | Trend of increased prevalence of acute rejection in CYP3A4*22 carriers compared to CYP3A4*1/*1 patients (37% vs. 28%, p=0.42). | (El-Shair et al., 2019) |  |
| Sirolimus | *In vitro* meta-bolism | 31 | Human liver microsomes | *CYP3A4*22* resulted in 20% lower metabolic rates (p=0.0411) of sirolimus *in vitro*. | (Woillard et al., 2013) |  |
| Everolimus | PK | 97 | Predominantly Caucasian adult renal transplant recipients | A trend of 7% lower everolimus clearance (p=0.39) in *CYP3A4*22* carriers compared to *CYP3A4*1/*1* patients. | (Moes et al., 2014) |  |
|  |  | 37 | Finish metastatic breast cancer patients | 2.7-fold higher everolimus blood concentrations (p=0.019) in *CYP3A4*22* carriers, compared to *CYP3A4*1/*1.* | (Pascual et al., 2017) |  |

**References**

Please see main article for references:
*Mulder TAM, van Eerden RAG, de With M, Elens L, Hesselink DA, Matic M, Bins S, Mathijssen RHJ and van Schaik RHN (2021) CYP3A4∗22 Genotyping in Clinical Practice: Ready for Implementation? Front. Genet. 12:711943. doi: 10.3389/fgene.2021.711943*
