# Supplementary material for: CYP3A4∗22 Genotyping in Clinical Practice: Ready for Implementation?
Source: Front Genet. 2021 Jul 8;12:711943. doi: 10.3389/fgene.2021.711943 (PMC8296839; doi:10.3389/fgene.2021.711943)
Supplement: Supplementary file 3 [file Table_3.docx]

Supplementary Table 3

*CYP3A4*22* Genotyping in Clinical Practice: Ready for Implementation?

*Tessa A.M. Mulder, Ruben A. G. van Eerden, Mirjam de With, Laure Elens, Dennis A. Hesselink, Maja Matic, Sander Bins, Ron H. J. Mathijssen and Ron H. N. van Schaik*

| **Supplementary Table 3: Summary of CYP3A4*22 influence on pharmacokinetics (PK), dose requirement (DR), or efficacy (Eff) of cardiovascular agents ticagrelor and sildenafil, and statins simvastatin and atorvastatin. Abbreviations: AUC: area under the plasma concentration-time curve.** | | | | | | |
| --- | --- | --- | --- | --- | --- | --- |
| **Drug class** | ***Drug*** | ***Effect*** | ***n=*** | ***Study population*** | ***Estimated change*** | ***Reference*** |
| Cardio-vascular agents | *Tica-grelor* | PK | 27 | Healthy Finnish white volunteers | 89% higher weight-adjusted ticagrelor AUC from 0 hours to infinity in *CYP3A4*22* carriers compared to wild-type patients (95% CI: 34-167, p=0.004). | (Holmberg et al., 2019) |
|  |  | Eff | 27 | Healthy Finnish white volunteers | *CYP3A4*22* carriers had a 43% platelet inhibitory effect at 24 hours after ticagrelor ingestion compared to a 21% inhibitory effect in wild-type patients (p=0.029) | (Holmberg et al., 2019 |
|  | *Clopi-dogrel* | PK | 27 | Healthy Finnish white volunteers | No significant influence of CYP3A4*22 on Cmax and AUCinf of clopidogrel and clopidogrel active metabolite. Significant influence of CYP3A4*22 on AUCinf of inactive clopidogrel carboxylic acid (p=0.026). | (Holmberg et al., 2019) |
|  | *Pra-sugrel* | PK | 27 | Healthy Finnish white volunteers | No significant influence of CYP3A4*22 on Cmax and AUCinf of prasugrel active metabolite and prasugrel inactive metabolite. | (Holmberg et al., 2019) |
|  | *Sil-denafil* | PK | 85 | Predominantly white patients with heart failure with preserved left ventricular ejection fraction | Significantly higher dose-adjusted peak concentrations of sildenafil in Caucasian *CYP3A4*22* carriers at week 12 and week 24 than wild type *CYP3A4* patients (adjusted p=0.0165 for repeated measures, no estimate change shown). | (de Denus et al., 2018) |
| Statines | *Sim-vastatin* | PK | three clinical trials n= 16 n= 18 n= 40 | Healthy Caucasian and Japanese volunteers | *CYP3A4*22* carriers had a 49% higher simvastatin bioavailability than wild-type patients (ANOVA p=0.086, likelihood ratio test p=0.019).  *Caution: frequency and height of simvastatin dose differed between studies.* | (Tsamandouras et al., 2014) |
|  |  |  | 830 | African- and Caucasian-Americans participating in the Cholesterol and Pharmacogenetics (CAP) clinical trial (total cholesterol 160-400 mg/dl) | Caucasian *CYP3A4*22* carriers had a 20% higher simvastatin plasma concentration and 14% higher simvastatin acid plasma concentration compared with wild-type patients (p=0.06 and p=0.04, respectively). *CYP3A5*3* allele status was not significantly associated with simvastatin or simvastatin acid concentrations. | (Kitzmiller et al., 2014) |
|  |  |  | 646 | African- and Caucasian-Americans participating in the Cholesterol and Pharmacogenetics (CAP) clinical trial (total cholesterol 160-400 mg/dl) | 58% increased plasma 12-hour concentration of simvastatin in *CYP3A4*22* carriers compared to wild-type patients (p=0.006). | (Luzum et al., 2015) |
|  |  | DR | 84 | Caucasians and African Americans taking stable doses of simvastatin | A multiple linear regression model, with total cholesterol level before treatment as covariate, showed that the stable simvastatin dose for *CYP3A4*22* carriers was a 0.6 fraction compared with wild-type patients (p=0.042). | (Wang et al., 2011) |
|  |  | Eff | 80 | Incident simvastatin users in Rotterdam study | *CYP3A4*22* carriers had a greater reduction in total (-0.31 mmol/l, p=0.028 ) and LDL (-0.34 mmol/l, p=0.034) cholesterol levels after adjustment for confounding factors, compared to wild-type patients. | (Elens et al., 2011a) |
|  | *Ator-vastatin* | PK | 56 | (German) healthy volunteers | CYP3A4*22 was independently associated with lower 2-OH-atorvastatin/atorvastatin AUC (p=0.001). Per copy of the *CYP3A4*22* allele the 2-hydroxyatorvastatin/atorvastatin AUCinf ratio was 35% lower as shown in a regression model. | (Klein et al., 2012) |
|  |  | DR | 142 | Caucasians and African Americans taking stable doses of atorvastatin | A multiple linear regression model, with total cholesterol level before treatment as covariate, showed that the stable atorvastatin dose for *CYP3A4*22* carriers was a 0.22 fraction compared with wild-type patients (p=0.024). | (Wang et al., 2011) |
|  | *Combi:*  *Sim-vastatinand*  *Ator-vastatin* | Eff | 416 | Greek patients with primary hyper-cholesterolemia | After 6 months, total cholesterol was decreased more in CYP3A4*22 carriers compared to CYP3A4*1/*1 (-37.1±9.5% vs.  -33.7±12.1%)(p>0.05). The levels of LDL cholesterol showed the same trend  (-49.8±13.6% vs. -42.4±20.4%)(all p>0.05)  CYP3A4*22 was also not significantly associated with percent reduction in cholesterol levels stratified per statin treatment. | (Ragia et al., 2015) |

**References**

Please see main article for references:
*Mulder TAM, van Eerden RAG, de With M, Elens L, Hesselink DA, Matic M, Bins S, Mathijssen RHJ and van Schaik RHN (2021) CYP3A4∗22 Genotyping in Clinical Practice: Ready for Implementation? Front. Genet. 12:711943. doi: 10.3389/fgene.2021.711943*
