# Supplementary material for: CYP3A4∗22 Genotyping in Clinical Practice: Ready for Implementation?
Source: Front Genet. 2021 Jul 8;12:711943. doi: 10.3389/fgene.2021.711943 (PMC8296839; doi:10.3389/fgene.2021.711943)
Supplement: Supplementary file 4 [file Table_4.docx]

Supplementary Table 4

*CYP3A4*22* Genotyping in Clinical Practice: Ready for Implementation?

*Tessa A.M. Mulder, Ruben A. G. van Eerden, Mirjam de With, Laure Elens, Dennis A. Hesselink, Maja Matic, Sander Bins, Ron H. J. Mathijssen and Ron H. N. van Schaik*

| **Supplementary Table 4: Summary of CYP3A4*22 influence on pharmacokinetics (PK) and clinical outcome of alprazolam. Abbreviations: HAMA: Hamilton Anxiety Rating Scale.** | | | | |
| --- | --- | --- | --- | --- |
| **Effect** | ***n=*** | ***Study population*** | ***Estimated change*** | ***Reference*** |
| PK | 105 | (Russian) patients with anxiety disorders comorbid with alcohol use disorder | *CYP3A4*22* carriers had significantly increased alprazolam concentration/dose ratios compared to wild-type patients (2.888 vs 1.583, p=0.001). | (Zastrozhin et al., 2020) |
| Clinical Outcome | 105 | (Russian) patients with anxiety disorders comorbid with alcohol use disorder | *CYP3A4*22* carriers had a significantly decreased treatment response as reflected in the HAMA scale scores compared to wild-type patients (*CYP3A4*1/*22*: 4.0 vs CYP3A4*1/*1: 3.0, p=0.007). | (Zastrozhin et al., 2020) |

**References**

Please see main article for references:
*Mulder TAM, van Eerden RAG, de With M, Elens L, Hesselink DA, Matic M, Bins S, Mathijssen RHJ and van Schaik RHN (2021) CYP3A4∗22 Genotyping in Clinical Practice: Ready for Implementation? Front. Genet. 12:711943. doi: 10.3389/fgene.2021.711943*
