# Supplementary material for: CYP3A4∗22 Genotyping in Clinical Practice: Ready for Implementation?
Source: Front Genet. 2021 Jul 8;12:711943. doi: 10.3389/fgene.2021.711943 (PMC8296839; doi:10.3389/fgene.2021.711943)
Supplement: Supplementary file 5 [file Table_5.docx]

Supplementary Table 5

*CYP3A4*22* Genotyping in Clinical Practice: Ready for Implementation?

*Tessa A.M. Mulder, Ruben A. G. van Eerden, Mirjam de With, Laure Elens, Dennis A. Hesselink, Maja Matic, Sander Bins, Ron H. J. Mathijssen and Ron H. N. van Schaik*

| **Supplementary Table 5: Summary of CYP3A4*22 influence on pharmacokinetics of anti-psychotics risperidone, aripiprazole, haloperidol, pimozide and quetiapine.** | | | | |
| --- | --- | --- | --- | --- |
| **Drug** | ***N=*** | ***Study population*** | ***Estimated change*** | ***Reference*** |
| Risperidone | 150 | Predominantly Caucasian psychiatric patients treated with risperidone | *CYP3A4*22* carriers had 30% lower mean apparent 9-hydroxyrisperidone clearance compared to non-carriers (p=0.008) | (Vandenberghe et al., 2015) |
|  | 396 | Predominantly white patients with psychotic disorders | No significant difference in serum levels of risperidone or hydroxyrisperidone were found between *CYP3A4*22* carriers and wild-type patients (5 vs 5 µg/L, and 15 vs 15 µg/L, respectively). No significant increase in C/D risperidone and hydroxyrisperidone were found between *CYP3A4*22* carriers and wild-type patients (3 vs. 2.9, and 5.8 vs. 5.6, respectively) (all p>0.05). | (van der Weide and van der Weide, 2015) |
|  | 64 | Caucasian pediatric patients using second-generation antipsychotics | No significant increase in C/D risperidone between poor CYP3A metabolizers [CYP3A4*1/*22+CYP3A5*3/*3] and extensive CYP3A metabolizers [CYP3A4*1/*1+CYP3A5*1 carriers] (delta C/D: 2.51, p-0.191) | (Rafaniello et al., 2018) |
| Aripiprazole | 130 | Predominantly white patients with psychotic disorders | No significant decrease in serum levels aripiprazole and dehydroaripiprazole were found between CYP3A4*22 carriers and wild-type patients (130 vs. 160 µg/L, and 50 vs. 60 µg/L, respectively). No significant decrease in C/D aripiprazole and dehydroaripiprazole were found between CYP3A4*22 carriers and wild-type patients (10 vs. 11, and 3.6 vs. 3.3, respectively) (all p>0.05). | (van der Weide and van der Weide, 2015) |
|  | 26 | Caucasian pediatric patients using second-generation antipsychotics | No significant decrease in C/D aripiprazole between poor CYP3A metabolizers [CYP3A4*1/*22+CYP3A5*3/*3] and extensive CYP3A metabolizers [CYP3A4*1/*1+CYP3A5*1 carriers] (delta C/D: -19.66, p=0.345). | (Rafaniello et al., 2018) |
| Haloperidol | 312 | Predominantly white patients with psychotic disorders | No significant decrease in haloperidol serum levels was found between CYP3A4*22 carriers and wild-type patients (1.85 vs. 2 µg/L). No significant decrease in C/D was found between CYP3A4*22 carriers and wild-type patients (0.8 vs. 0.9) (all p>0.05). | (van der Weide and van der Weide, 2015) |
| Pimozide | 86 | Predominantly white patients with psychotic disorders | Significant decrease in serum levels pimozide was found between *CYP3A4*22* carriers and wild-type patients (1 vs. 3.4 µg/L, p<0.05)  Multiple regression analysis showed that *CYP3A4*22* genotype was significantly associated with serum levels pimozide and C/D ratio. However, this association explained only 5% of the total variation. | (van der Weide and van der Weide, 2015) |
| Quetiapine | 238 | Predominantly white psychiatric patients receiving quetiapine | *CYP3A4*22* carriers had 2.5-fold higher serum levels of quetiapine compared to wild-type patients (p=0.03) when using a similar dose (median both groups= 300mg/day, p=0.67). *CYP3A4*22* carriers had 67% higher dose-corrected serum concentrations than wild-type patients (p=0.01). A higher number of *CYP3A4*22* patients achieved serum levels above the therapeutic range of 500 µg/L, compared to wild-type patients (16.1% vs. 2.9%, p=0.007). | (van der Weide and van der Weide, 2014) |

**References**

Please see main article for references:
*Mulder TAM, van Eerden RAG, de With M, Elens L, Hesselink DA, Matic M, Bins S, Mathijssen RHJ and van Schaik RHN (2021) CYP3A4∗22 Genotyping in Clinical Practice: Ready for Implementation? Front. Genet. 12:711943. doi: 10.3389/fgene.2021.711943*
