# Supplementary material for: CYP3A4∗22 Genotyping in Clinical Practice: Ready for Implementation?
Source: Front Genet. 2021 Jul 8;12:711943. doi: 10.3389/fgene.2021.711943 (PMC8296839; doi:10.3389/fgene.2021.711943)
Supplement: Supplementary file 6 [file Table_6.docx]

Supplementary Table 6

*CYP3A4*22* Genotyping in Clinical Practice: Ready for Implementation?

*Tessa A.M. Mulder, Ruben A. G. van Eerden, Mirjam de With, Laure Elens, Dennis A. Hesselink, Maja Matic, Sander Bins, Ron H. J. Mathijssen and Ron H. N. van Schaik*

| **Supplementary Table 6: Summary of CYP3A4*22 influence on pharmacokinetics (PK), dose requirement (DR), or toxicity (Tox) of different anti-cancer drugs. Abbreviations: AUC: area under the plasma concentration-time curve, Cl: Clearance, DR: required dose** | | | | | | |
| --- | --- | --- | --- | --- | --- | --- |
| **Drug class** | ***Drug*** | ***Effect*** | ***N=*** | ***Study population*** | ***Estimated change*** | ***Ref.*** |
| ER-positive breast cancer | *Tam-oxifen* | PK | 178 | Predominantly white woman on adjuvant hormonal tamoxifen treatment (20 mg/day) | *CYP3A4*22* carriers had a 47% increase of median 4-hydroxytamoxifen concentration compared with the wild-type patients (1.71 vs 1.16 ng/ml, p<0.05). *CYP3A4*22* carriers also had a 53% increase in tamoxifen concentration compared with the wild-type patients (130.82 vs 85.05 ng/ml, p<0.05). Higher [tamoxifen]/[n-desmethyltamoxifen] metabolic ratio (0.72 vs 0.52, p<0.05) and higher [hydroxytamoxifen]/[endoxifen] metabolic ratio (0.18 vs 0.14, p=0.06) was observed in *CYP3A4*22* carriers compared to wild-type patients. | (Antunes et al., 2015) |
|  |  |  | 196 | Predominantly Caucasian patients on adjuvant tamoxifen therapy | *CYP3A4*22* carriers had higher levels of tamoxifen (p<0.0001), NDM-tamoxifen (p<0.0001), 4-OH-tamoxifen (p<0.001) and endoxifen (p<0.05) compared to *CYP3A4*1/*1* patients. Less *CYP3A4*22* carriers had endoxifen levels below sub-therapeutic range compared to *CYP3A4*1/*1* patients (10% vs. 23%). *CYP3A4*22* carriers had lower metabolic ratio of total metabolite (NDM-tamoxifen + 4-OH-tamoxifen + endoxifen) : tamoxifen compared to *CYP3A4*1/*1* patients (p<0.05).  In all CYP2D6 genotype defined groups, increased endoxifen levels in *CYP3A4*22* carriers compare to *CYP3A4*1/*1* patients were defined. | (Teft et al., 2013) |
|  |  |  | 132 | Predominantly Caucasian breast cancer patients on tamoxifen therapy | *CYP3A4*22* carriers had higher endoxifen concentration than wild-type patients (p<0.05) | (Baxter et al., 2014) |
|  |  | Tox | 132 | Predominantly Caucasian breast cancer patients on tamoxifen therapy | *CYP3A4*22* carriers were significantly less likely to experience hot flashes (odds ratio 8.87, p<0.01), even when compared to patients with similar endoxifen levels.  CYP3A4*22 carriers had higher endoxifen concentration than wild-type patients (p<0.05) | (Baxter et al., 2014) |
|  | *Exe-mes-tane* | PK | 246 | Predominantly white postmenopausal breast cancer patients | 54% higher exemestane concentrations were found in *CYP3A4*22* carriers as compared to *CYP3A4* wild-type patients (p<0.01). CYP3A4*22 maintained significantly associated after adjustment for covariates (p<0.01). | (Hertz et al., 2017) |
| Micro-tubule-stabi-lizing agent | *Pac-litaxel* | Tox | 261 | Predominantly Caucasian patients with cancer treated with paclitaxel for different tumor types | Female *CYP3A4*22* carriers had increased risk of developing neurotoxicity (p=0.043). *CYP3A4*22* allele status itself was not associated with pharmacokinetic parameters (*e.g*. Cl and AUC). In the validation cohort, *CYP3A4*22* carriers were at risk of developing grade 3 neurotoxicity (OR=19.1, p=0.001). | (Graan et al., 2013) |
|  | *Doce-taxel* | Tox | 150 | (Swedish) HER2-negative breast cancer patients in the PROMIX trial | The number of reported grade 3-4 adverse events differed between phenotype groups (p=0.02), with highest risk in the poor metabolizer group [CYP3A4*22 carriers+CYP3A5*3/*3]. During the whole treatment period, 71% of the poor metabolizers experienced grade 3-4 adverse events, compared to 54% in intermediate metabolizers [CYP3A4*1/*1+CYP3A5*3/*3] and 10% in extensive metabolizers [CYP3A4*1/*1+CYP3A5*1 carriers](p=0.57). | (Sim et al., 2018) |
| Tyrosine kinase inhi-bitors | *Pazo-panib* | PK | three studies n=40 n=41 n-13 | Predominantly Caucasian patients diagnosed with advanced solid tumors or metastatic renal cell cancer using pazopanib | *CYP3A4*22* carriers had a 35% decreased Cl of pazopanib compared to wild-type patients (0.18 vs. 0.27, p<0.005). | (Bins et al., 2019) |
|  |  | DR | three studies n=40 n=41 n=13 | Predominantly Caucasian patients diagnosed with advanced solid tumors or metastatic renal cell cancer using pazopanib | Simulated median trough concentrations of *CYP3A4*22* carriers using 600 mg once daily were 31 mg/L compared to 18 mg/L in wild-type patients. Simulated median trough concentration of *CYP3A4*22* carriers using 800 mg once daily were 35 mg/L, compared to 20 mg/L in wild-type patients. (no p-values, because results were not compared). | (Bins et al., 2019) |
|  | *Suni-tinib* | PK | 114 | Predominantly Caucasian patients with different solid tumors treated with sunitinib | *CYP3A4*22* status was associated with a 22.5% decreased Cl of sunitinib (p<0.01). Note: The observed effect sizes are below the interindividual variability in clearance. (threshold p-value due to multiple testing= 0.0005) | (Diekstra et al., 2014) |

**References**

Please see main article for references:
*Mulder TAM, van Eerden RAG, de With M, Elens L, Hesselink DA, Matic M, Bins S, Mathijssen RHJ and van Schaik RHN (2021) CYP3A4∗22 Genotyping in Clinical Practice: Ready for Implementation? Front. Genet. 12:711943. doi: 10.3389/fgene.2021.711943*
