# Supplementary material for: CYP3A4∗22 Genotyping in Clinical Practice: Ready for Implementation?
Source: Front Genet. 2021 Jul 8;12:711943. doi: 10.3389/fgene.2021.711943 (PMC8296839; doi:10.3389/fgene.2021.711943)
Supplement: Supplementary file 7 [file Table_7.docx]

Supplementary Table 7

*CYP3A4*22* Genotyping in Clinical Practice: Ready for Implementation?

*Tessa A.M. Mulder, Ruben A. G. van Eerden, Mirjam de With, Laure Elens, Dennis A. Hesselink, Maja Matic, Sander Bins, Ron H. J. Mathijssen and Ron H. N. van Schaik*

| **Supplementary Table 7: Summary of CYP3A4*22 influence on pharmacokinetics of fentanyl. Abbreviations: AUC: area under the plasma concentration-time curve, Cl: Clearance.** | | | |
| --- | --- | --- | --- |
| **n=** | ***Study population*** | ***Estimated change*** | ***Reference*** |
| 35 | Caucasian and Latin-American healthy volunteers | *CYP3A4*22* carriers showed higher AUC (p=0.002) and lower Cl (p=0.015) compared to wild-type patients. | (Saiz-Rodríquez et al., 2019) |
| 251 | Predominantly Caucasian and Latin-American healthy volunteers | Individuals carrying *CYP3A4* variant alleles *3, *20, *22 showed a trend of higher normalized AUC (p=0.099) and lower normalized Cl (p=0.069). | (Saiz-Rodríquez et al., 2020) |
| 676 | European cancer patients with malignant disease treated with transdermal fentanyl | *CYP3A4*22* carrier status was associated with serum norfentanyl concentration (coefficient estimate = -0.23, p=0.007), but not with serum fentanyl concentration (no p-value).  *CYP3A4*22* carrier status was associated with serum norfentanyl : fentanyl concentration ratios (coefficient estimate=-0.31, p=0.029). | (Barratt et al., 2014) |

**References**

Please see main article for references:
*Mulder TAM, van Eerden RAG, de With M, Elens L, Hesselink DA, Matic M, Bins S, Mathijssen RHJ and van Schaik RHN (2021) CYP3A4∗22 Genotyping in Clinical Practice: Ready for Implementation? Front. Genet. 12:711943. doi: 10.3389/fgene.2021.711943*
