# Supplementary material for: CYP3A4∗22 Genotyping in Clinical Practice: Ready for Implementation?
Source: Front Genet. 2021 Jul 8;12:711943. doi: 10.3389/fgene.2021.711943 (PMC8296839; doi:10.3389/fgene.2021.711943)
Supplement: Supplementary file 8 [file Table_8.docx]

Supplementary Table 8

*CYP3A4*22* Genotyping in Clinical Practice: Ready for Implementation?

*Tessa A.M. Mulder, Ruben A. G. van Eerden, Mirjam de With, Laure Elens, Dennis A. Hesselink, Maja Matic, Sander Bins, Ron H. J. Mathijssen and Ron H. N. van Schaik*

| **Supplementary Table 8: Summary of CYP3A4*22 influence on pharmacokinetics (PK) and dose requirement (DR) of anti-viral drugs lopinavir and tenofovir alafenamide. Abbreviations: AUC: area under the plasma concentration-time curve** | | | | | |
| --- | --- | --- | --- | --- | --- |
| **Drug** | ***Effect*** | ***n=*** | ***Study population*** | ***Estimated change*** | ***Reference*** |
| Lopinavir | PK | 375 | HIV-positive patients using 400/100 mg lopinavir/ritonavir  (no ethnic information) | Patient genotyped as *CYP3A4*22/*22* had lower simulated lopinavir clearance compared to CYP3A4*1/*1 patients (p=0.028), and also lower lopinavir clearance compared to CYP3A4*1/*22 carriers (p=0.023). A trend of lower lopinavir clearance in CYP3A4*1/*22 compared to CYP3A4*1/*1 was shown (p=0.254). *CYP3A4*22/*22* patients showed a 2.3-fold higher lopinavir trough concentration compared with wild-type *CYP3A4* patients (no p-value shown).  Patients genotyped as [CYP3A4*22/*22, SLCO1B1 521T>C/521T>C] had a 9.7-fold higher lopinavir trough concentration compared with wild-type patients (no p-values shown). | (Olagunju et al., 2014) |
|  | DR | 375 | HIV-positive patients using 400/100 mg lopinavir/ritonavir (no ethnic information) | Simulated dosage reduction scenarios showed that individuals genotyped as CYP3A4*22/*22 and [*CYP3A4*22/*22, SLCO1B1 521CC*] were predicted to achieve a mean lopinavir trough concentration of 4740 ng/ml (95% CI 1650-10.860) and 20,130 ng/ml (95% CI: 6930-47,930 ng/ml), respectively. Both are well above minimum effective concentration of 1000 ng/ml. | (Olagunju et al., 2014) |
| Tenofovir alafenamide | PK | 21 | Healthy volunteers (no ethnic information) | *CYP3A4*22* carriers had a 39% higher plasma tenofovir alafenamide AUC_0-24_ at day 56 compared to non-carriers (p=0.033). | (Cerrone et al., 2019) |

**References**

Please see main article for references:
*Mulder TAM, van Eerden RAG, de With M, Elens L, Hesselink DA, Matic M, Bins S, Mathijssen RHJ and van Schaik RHN (2021) CYP3A4∗22 Genotyping in Clinical Practice: Ready for Implementation? Front. Genet. 12:711943. doi: 10.3389/fgene.2021.711943*
